# Conditional Ablation of the Choroideremia Gene Causes Age-Related Changes in Mouse Retinal Pigment Epithelium

**DOI:** 10.1371/journal.pone.0057769

**Published:** 2013-02-27

**Authors:** Silène T. Wavre-Shapton, Tanya Tolmachova, Mafalda Lopes da Silva, Clare E. Futter, Miguel C. Seabra

**Affiliations:** 1 Molecular Medicine Section, National Heart and Lung Institute, Imperial College London, London, United Kingdom; 2 UCL Institute of Ophthalmology, University College London, London, United Kingdom; 3 CEDOC, Faculdade de Ciências Médicas, Universidade Nova de Lisboa, Lisbon, Portugal; 4 Instituto Gulbenkian de Ciência, Oeiras, Portugal; University of Florida, United States of America

## Abstract

The retinal pigment epithelium (RPE) is a pigmented monolayer of cells lying between the photoreceptors and a layer of fenestrated capillaries, the choriocapillaris. Choroideremia (CHM) is an X-linked progressive degeneration of these three layers caused by the loss of function of Rab Escort protein-1 (REP1). REP1 is involved in the prenylation of Rab proteins, key regulators of membrane trafficking. To study the pathological consequences of chronic disruption of membrane traffic in the RPE we used a cell type-specific knock-out mouse model of the disease, where the *Chm/Rep1* gene is deleted only in pigmented cells (*Chm^Flox^, Tyr-Cre+*). Transmission electron microscopy (TEM) was used to quantitate the melanosome distribution in the RPE and immunofluorescent staining of rhodopsin was used to quantitate phagocytosed rod outer segments in retinal sections. The ultrastructure of the RPE and Bruch’s membrane at different ages was characterised by TEM to analyse age-related changes occurring as a result of defects in membrane traffic pathways. *Chm/Rep1* gene knockout in RPE cells resulted in reduced numbers of melanosomes in the apical processes and delayed phagosome degradation. In addition, the RPE accumulated pathological changes at 5–6 months of age similar to those observed in 2-year old controls. These included the intracellular accumulation of lipofuscin-containing deposits, disorganised basal infoldings and the extracellular accumulation of basal laminar and basal linear deposits. The phenotype of the *Chm^Flox^, Tyr-Cre+* mice suggests that loss of the *Chm/Rep1* gene causes premature accumulation of features of aging in the RPE. Furthermore, the striking similarities between the present observations and some of the phenotypes reported in age-related macular degeneration (AMD) suggest that membrane traffic defects may contribute to the pathogenesis of AMD.

## Introduction

The retinal pigment epithelium (RPE) provides nutrients, growth factors and ions to the photoreceptors, removes waste products of retinal metabolism and is essential for photoreceptor survival and, hence, for vision. RPE dysfunction is associated with aging and multiple inherited retinal degenerative diseases. One such disease, choroideremia (CHM), is an X-linked chorioretinal degeneration caused by functional defects in *CHM/REP1,* a chaperone protein for Rab GTPases [1], which are critical regulators of membrane trafficking [2]. Loss of function of Rab Escort Protein-1 (REP1) in CHM results in reduced Rab GTPase prenylation, a lipid modification that is absolutely required for Rab membrane binding and function [1]. Loss of function of REP1 in CHM is functionally compensated by a related protein, REP2 [3]. However, this compensation is only partial as a subset of Rabs are underprenylated in peripheral lymphoblasts of CHM patients and in mouse models of CHM [4], [5]. Given that Rab GTPases regulate multiple steps in membrane traffic pathways including vesicle budding, movement and fusion with the destination compartment, the partial loss of function of multiple Rabs is predicted to affect multiple intracellular trafficking pathways. One of the partially affected Rabs in CHM is Rab27a, which is required for melanosome movement into the apical processes of RPE cells [6], [7]. However the pathology of CHM cannot be explained solely by compromised Rab27a function as the *ashen* mouse, which lacks functional Rab27a, does not reproduce the retinal degeneration observed in CHM patients or in CHM mouse models [4], [7].

Consistent with multiple trafficking defects in the development of CHM, reduced lysosomal acidification and secretion of cytokines have been detected in monocytes isolated from CHM patients [8] and there is reduced melanosome movement into the apical processes of RPE cells in mouse models of CHM [9]. Furthermore cultured RPE cells acutely depleted of REP1 exhibit reduced lysosome acidification and delayed phagosome degradation [10]. Despite the demonstration of both underprenylated Rabs and trafficking defects in peripheral cells, the pathological features of CHM are restricted to the eye and characterised by progressive degeneration of photoreceptors, RPE and choroid in patients with CHM, leading ultimately to blindness. The particular susceptibility of the eye to loss of REP1 is not completely understood. Both the photoreceptors and the RPE are largely postmitotic and have a huge traffic burden from the daily production of outer segments by the photoreceptors and the daily degradation of phagocytosed shed outer segments by the RPE, which may render these cell types particularly susceptible to partial defects in membrane traffic.

In this study we use a CHM mouse model previously described where, in addition to the conditional allele of the *Chm/Rep1* gene, mice carry the *Cre*-transgene under the control of the tyrosinase promoter (*Tyr-Cre*) which leads to a knock-out of the *Chm/Rep1* gene only in pigmented cells [9]. These *Chm^Flox^, Tyr-Cre+* mice have enabled us to study the long-term consequences of chronic membrane traffic defects in the RPE.

## Materials and Methods

### Mice

All animals used in this study were treated humanely in accordance with Home Office guidance rules under project licence 70/6176 and 70/7078, adhering to the ARVO Statement for the Use of Animals in Ophthalmic and Vision Research. The conditional knock-out mouse line *Chm^Flox^,Tyr-Cre*, that carries the *Cre*-recombinase transgene under control of the tyrosinase promoter, was generated previously and genotyping of mice was performed as described [9]. As controls we used *Chm^Flox^* littermates and *Chm^+^*(WT) mice on the C57Bl6 background. Both females and males were used in this study. Rab27a-mutant (*ashen, Rab27^ash/ash^*) mice were purchased from Jackson Laboratory (Bar Harbor, Maine, USA) and bred onto a C57Bl6 background.

### Immuofluorescence Analysis

Eyes were fixed in 4% paraformaldehyde in PBS for 2 hours at room temperature or overnight at 4°C, incubated overnight at 4°C in 30% sucrose in PBS and embedded in Tissue Tec. 10-µm thick sections were cut at −20°C and dried at room temperature. Sections were immunolabelled with anti-Rhodopsin (RetP1, from Abcam, UK) antibody followed by secondary antibody conjugated to Alexa^488^ (from Molecular Probes, UK). Eyecups were cut parallel and adjacent to the optic nerve in order to visualise peripheral and central retina. Samples were analysed by confocal microscopy (Leica TCS-SP2) and the number of phagosomes per length of RPE was counted manually. To eliminate autofluorescence from our analysis, microscope settings were adjusted so that negative controls, where primary antibodies were omitted, were negative. Between 3 and 5 animals were analysed in each group.

### Transmission Electron Microscopy

Eyes were fixed for 90 minutes at room temperature or overnight at 4°C in 2% paraformaldehyde and 2% glutaraldehyde in 0.1M cacodylate buffer. The cornea was cut off to remove the lens. The eyes were then postfixed in 1.5% potassium ferricyanide and 1% Osmium tetroxide for 2 hours on ice. The eyes were then dehydrated in ethanol (70%, 90% and absolute) and propylene oxide and transferred to 1∶1 propylene oxide:Epon overnight followed by two changes and embedding in Epon. Ultra-thin sections were stained with lead citrate before examination. As for light microscopy, eyecups were cut parallel and adjacent to the optic nerve in order to visualise peripheral and central retina. Samples were viewed on a JEOL 1010 transmission electron microscope (Welwyn Garden City, United Kingdom). Images were taken with a Gatan Orius SC100B charge-coupled device camera and analysed with Gatan Digital Micrograph and Adobe Photoshop.

### Quantification

For quantification of the number of melanosomes, lipofuscin granules and melanolipofuscin by conventional EM, organelles were scored in at least 200 µm length of RPE. A minimum of 4 animals were analysed in each group. To quantify the percentage of melanosomes in the apical processes, the position of the melanosomes was recorded as apical if above the tight junctions. For quantification of Bruch’s membrane (BrM) thickness, 10 images were taken at regular intervals along sections of the entire eyecup, thus including peripheral and central retina. In each image the average of 5 measurements of the thickness of BrM was calculated. Four *Chm^Flox^, Tyr-Cre+* mice and their littermate controls were analysed. For quantification of basal laminar deposits (BLamDs) between 1.5 and 2 mm of length of RPE for each eye were analysed. The length of RPE containing BLamDs was measured on 11 *Chm^Flox^, Tyr-Cre+* mice and 14 *Chm^Flox^ control* mice.

### Statistics

To determine the significance of the data the non-parametric Mann and Whitney test was used throughout. A *P* value under 0.05 was considered statistically significant.

## Results

### Loss of Rep1 in the RPE causes Defects in Membrane Traffic Pathways *in vivo*

**Figure 1:**
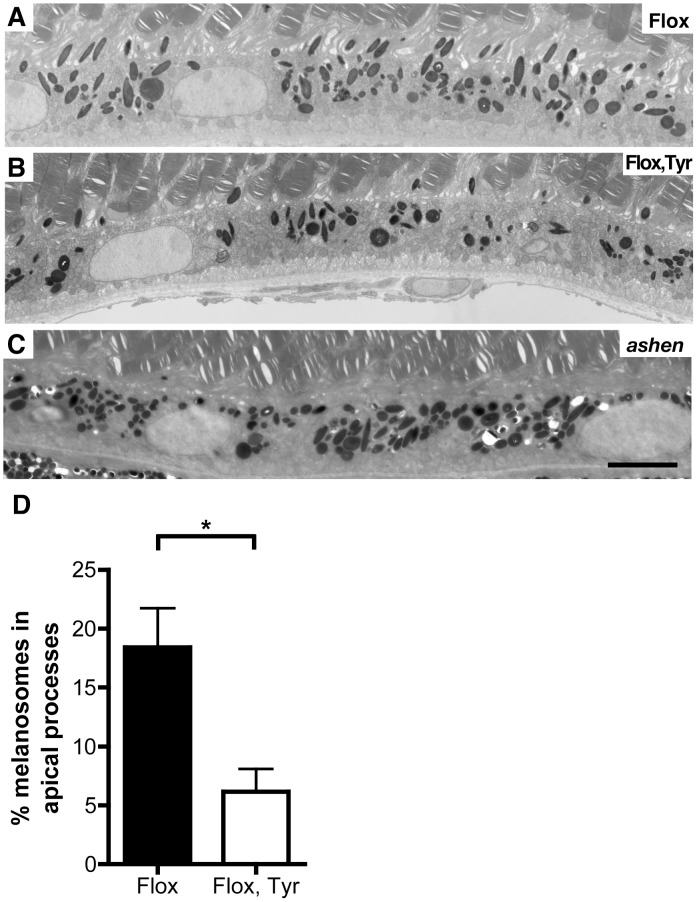
Impaired melanosome distribution in *Chm^Flox^*, *Tyr-Cre+* mice. Electron micrographs of the RPE of 7-month old *Chm^Flox^* (A), *Chm^Flox^, Tyr-Cre^+^* (B) and *ashen* (C) mice. Scale bars: 5 µm. The percentage of melanosomes in the apical processes in *Chm^Flox^* and *Chm^Flox^*, *Tyr-Cre+* mice was determined (D). No melanosomes were ever found in the apical processes of *ashen* mice. Results are mean+/−SEM of 5 to 6 observations. *P<0.05.

Our previous investigation of *Chm^Flox^, Tyr-Cre+* animals suggested a possible reduction in number of melanosomes in the apical processes of the RPE [9]. Here we have quantitated these effects and compared them to the *ashen* mouse, lacking functional Rab27a. The percentage of melanosomes found in the apical processes of the RPE in 7 to 12-month old *Chm^Flox^, Tyr-Cre+* mice 6 to 8 hours after light onset was reduced compared to littermate *Chm^Flox^* controls (Fig. 1A–D, 18.2% vs 6.2%, respectively), but did not show the complete exclusion from the apical processes that was exhibited by the *ashen* RPE (Fig. 1C). This reflects the partial nature of the prenylation defect in the *Chm^Flox^, Tyr-Cre*+ where a small amount of Rab27a is prenylated preventing a total block in melanosome movement. Similarly, melanosome movement in skin melanocytes is only partially affected in *Chm^Flox^, Tyr-Cre*+, as reflected by the mild coat colour defect [9]. No clear changes were observed in melanosome distribution in uveal melanocytes (as shown in Fig. S1). In addition no obvious changes in the number of melanosomes and/or melanocytes in the choroid of *Chm^Flox^, Tyr-Cre*+ were observed. In both *Chm^Flox^, Tyr-Cre*+ and littermate control mice choroidal melanosomes vary in size (Fig. S1C and S1D). Their distribution was not obviously affected by loss of REP1 but uveal melanocytes are extremely packed with melanosomes, making changes in their distribution difficult to analyse. It is worth noting that in the *ashen* mouse, that exhibits dramatic changes in melanosome distribution in the RPE [6], [7], no clear changes in uveal melanocytes can be observed.

**Figure 2:**
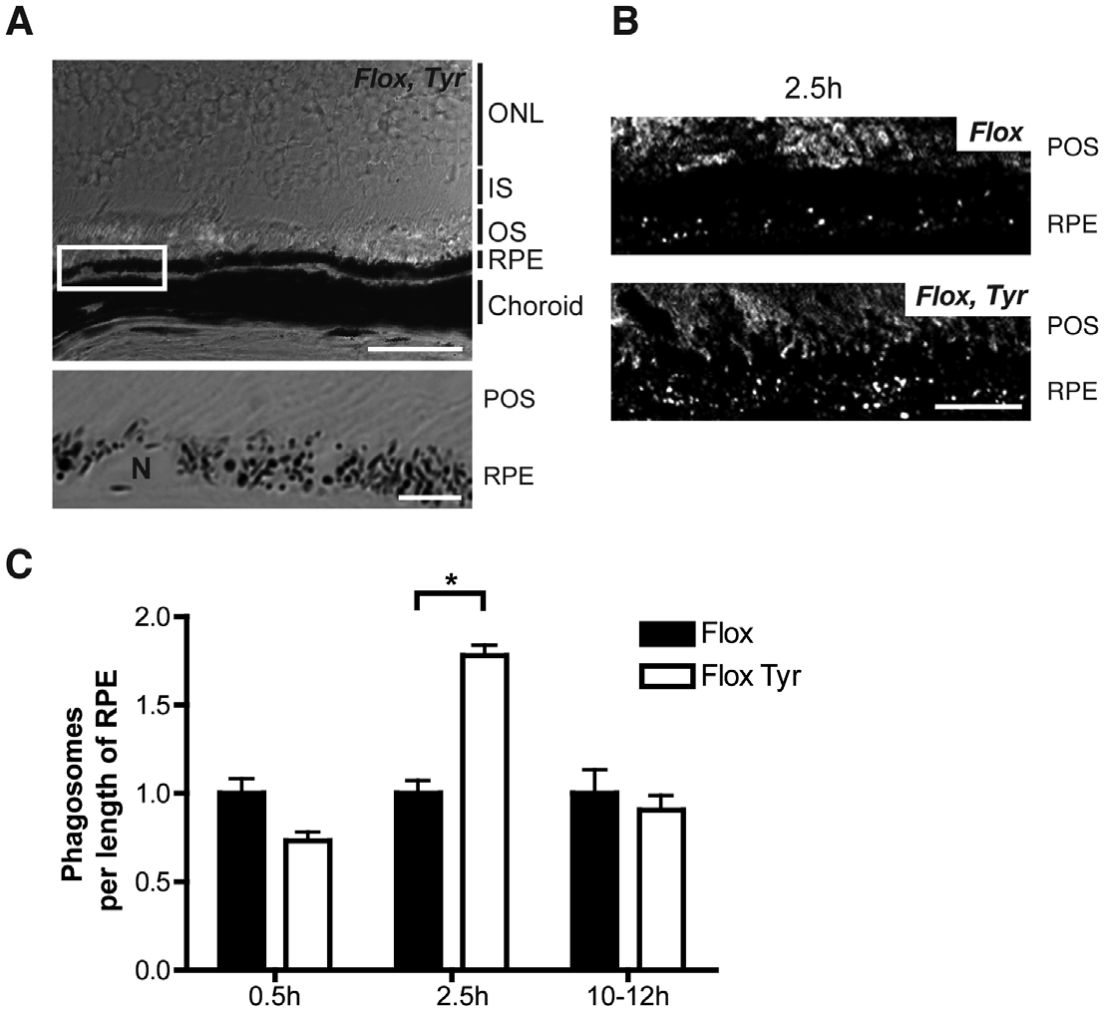
Increased number of phagosomes in *Chm^Flox^*, *Tyr-Cre+* mice. Frozen sections of eyes from 6-month old *Chm^Flox^*, *Tyr-Cre+* mice and littermates *Chm^Flox^* mice harvested at the indicated times after light onset were immunostained with antibody (RetP1) for rhodopsin and analysed by confocal microscopy. Phase images were used to identify the RPE within the tissue. (A) Overview of the choroid, RPE, and part of the retina in *Chm^Flox^*, *Tyr-Cre+* sample. Scale bar: 50 µm. The boxed region is magnified in the panel beneath and shows an area of the RPE with overlaying POS, scale bar: 5 µm. (B) Projections of 9 confocal sections. Scale bar: 10 µm. (C) Phagosomes were counted and results were normalised to the control and are presented as mean+/−SEM of 3–5 observations. *P<0.05. (ONL) Outer nuclear layer, (IS) Inner segment, (OS) Outer segment.

Delayed phagosome degradation has been demonstrated *in vitro* following acute depletion of REP1 [10]. To determine whether phagosome degradation was impaired *in vivo* following deletion of Rep1 in the RPE the number of phagosomes in the RPE was quantified using rhodopsin immunofluorescence of retinal sections (Fig. 2B). Phase images allowed the pigmented RPE to be readily identified in the tissue so that rhodopsin-positive phagosomes could be distinguished from the rest of the rhodopsin staining in the photoreceptor outer segment (POS) (Fig. 2A). The number of phagosomes 30 minutes after light onset was not significantly altered in 6-month old *Chm^Flox^, Tyr-Cre+* compared to littermate *Chm^Flox^* mice (Fig. 2C), suggesting that the early stages of phagosome uptake were not affected by loss of Rep1. The lengths of the POS were the same in control and mutant mice, also suggesting normal phagosome uptake which is in agreement with the results of Gordiyenko et al. [10] who showed no significant effects of REP1 depletion on phagosome uptake *in vitro*. However, 2.5 hours after light onset there were significantly more phagosomes in *Chm^Flox^, Tyr-Cre+* (1.75 fold more) than in littermate *Chm^Flox^*, indicating a delay in phagosome degradation (Fig. 2B, 2C). The same analysis 10–12 h after light onset showed that the majority of phagosomes had been degraded in both, *Chm^Flox^* and *Chm^Flox^, Tyr-Cre+*, so that there was little difference between the 2 samples (Fig. 2C).

### Loss of Rep1 in the RPE causes the Accumulation of Intracellular Deposits

**Figure 3:**
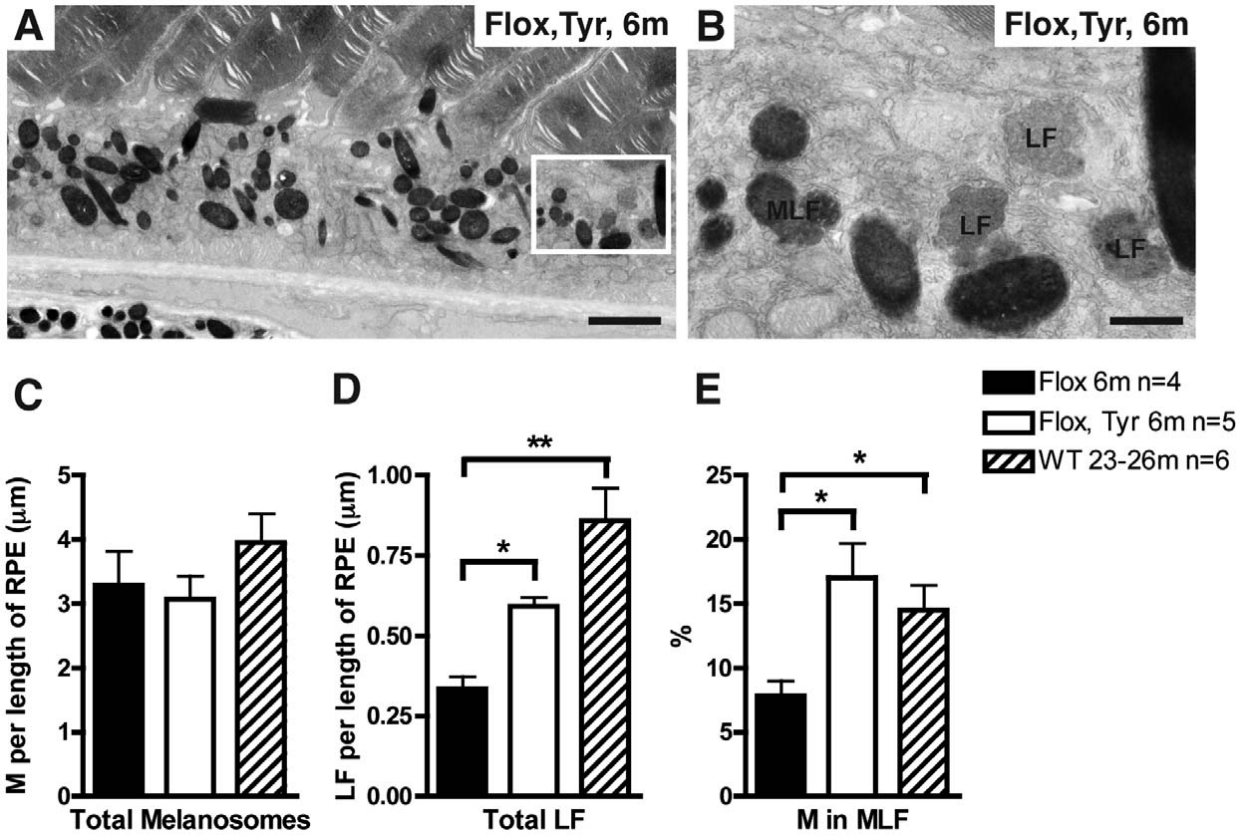
Accumulation of lipofuscin containing granules in *Chm^Flox^*, *Tyr-Cre+* mice. (A, B) Electron micrographs of 6-month old *Chm^Flox^*, *Tyr-Cre+* showing lipofuscin containing granules and melanolipofuscin. B is an enlargement of the area boxed in A. Scale bars: 2 µm (A), 0.5 µm (B). (C–E) Retinas of 6-month old *Chm^Flox^* mice (black bars), littermates *Chm^Flox^*, *Tyr-Cre+* (dark grey bars) and 2-year old control mice (light grey bars) were analysed for their content of melanosomes (M), lipofuscin containing granules (LF) and melanolipofuscin (MLF), respectively in the RPE. Melanosomes, lipofuscin containing granules and melanolipofuscin structures were counted and results are shown in the graphs per length of RPE (in µm) (for melanosomes and lipofuscin containing granules) or as a percentage of melanosomes associated with melanolipofuscin. Results are mean+/−SEM of at least 3 observations. *P<0.05, **P<0.01.

With time, lipofuscin containing granules (thought to be a degradative product of POS) accumulate in all aging RPE [11], [12]. Cytoplasmic deposits containing lipofuscin granules and melanin were observed in the cytoplasm of *Chm^Flox^, Tyr-Cre+* RPE as early as 2 months after birth (not shown). Quantitatively, the amount of lipofuscin containing granules was two-fold higher in 6-month old *Chm^Flox^, Tyr-Cre+* mice than in littermate *Chm^Flox^* and almost three-fold higher in 2-year old wild type mice (Fig. 3D). Lipofuscin containing granules were often associated with one or more melanosomes forming the so-called melanolipofuscin (Fig. 3B). Although the RPE of the *Chm^Flox^, Tyr-Cre+* mouse exhibited patchy depigmentation (Fig. 1B and Fig. S1B) overall there was not a significant reduction in melanosome number (Fig. 3C). However the percentage of these melanosomes that associated with lipofuscin granules more than doubled in 6-month old *Chm^Flox^, Tyr-Cre+* and 2-year old wild type mice compared with 6-month old control mice (Fig. 3E), indicating that association of melanosomes with lipofuscin granules is a feature of aging in the RPE and is accelerated by the loss of Rep1.

### Loss of Rep1 in the RPE causes Accumulation of Extracellular Deposits

**Figure 4:**
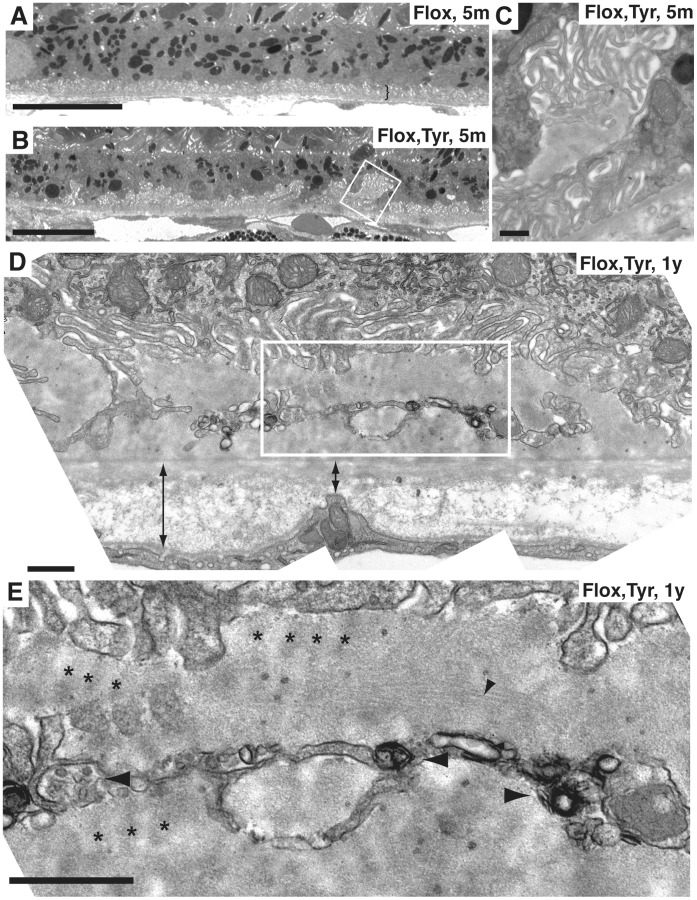
Irregularity of basal infoldings and basal laminar deposits in *Chm^Flox^*, *Tyr-Cre+* mice. Electron micrographs of the RPE of 5-month old *Chm^Flox^* (A), littermate *Chm^Flox^, Tyr-Cre^+^* (B and C) and 1-year old *Chm^Flox^*, *Tyr-Cre^+^* (D–E) mice. BIs are very regular in control mice (parenthesis in A). They disappear in some areas or expand in the cytoplasm of *Chm^Flox^*, *Tyr-Cre^+^* mice (B). The box in B is enlarged in panel C and shows early BLamDs underneath BIs. Membrane debris and membrane bound vesicles accumulate in late BLamDs (D and E). Panel E is a magnification of the rectangular box in D. Note thickening of BrM in D. Small arrowheads indicate fibrillar materials in BLamDs, asterisks highlight striations, big arrowheads indicate membrane debris, double arrows show BrM thickness. Scale bars: 10 µm (A, B), 0.5 µm (C–E).

**Figure 5:**
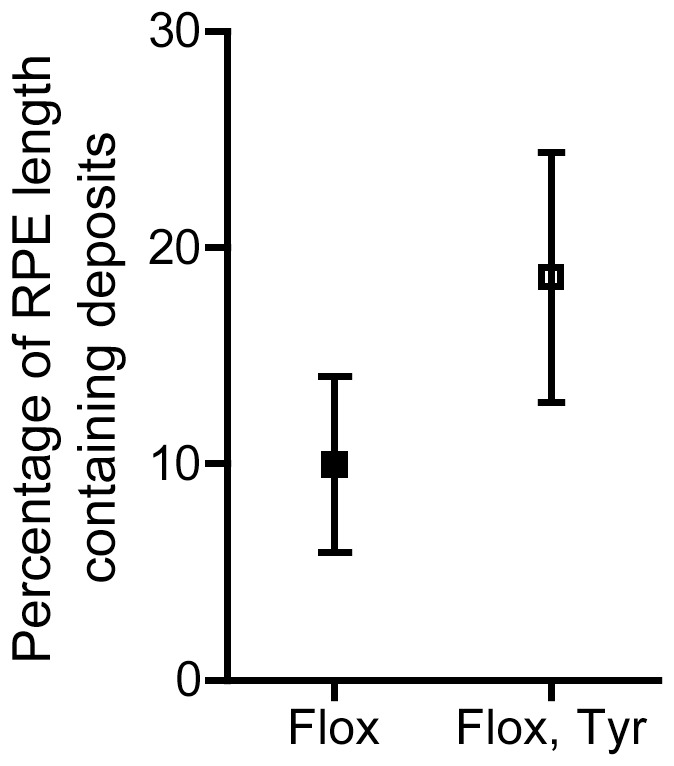
Quantification of basal laminar deposits. Length of RPE containing BLamDs was measured in 11 *Chm^Flox^*, *Tyr-Cre+* (black square) and 14 littermate control mice (white square) aged between 5 and 13 months. Graph shows percentage of RPE length containing deposits. Results are mean +/−SEM.

Further analysis by TEM of the morphology of the RPE of 5-month old *Chm^Flox^, Tyr-Cre+* eyes revealed disorganisation of the basal infoldings (BIs) such that they were absent in some areas and extended far into the cytoplasm of the RPE cells in other areas (Fig. 4B and 4C). In contrast, the thickness and organisation of the BIs was regular throughout the eyecup of age-matched wild type or littermate *Chm^Flox^* eyes (Fig. 4A). Additionally, fibrillar materials were observed between the basal membrane of the RPE and the basal lamina [Basal laminar Deposits (BlamDs)] more frequently in *Chm^Flox^, Tyr-Cre+* mice than in *Chm^Flox^* controls (Fig. 4C). Late BLamDs had more defined striations, resembling banded collagen type VI [13], observed in the eyes of AMD [14], Sorsby’s fundus dystrophy patients [15] and CHM carriers [16]. These deposits also contained membrane debris and vesicles. They were observed less frequently in 5-month old *Chm^Flox^, Tyr-Cre+* mice but were common by 1-year (Fig. 4D–E). The deposits were measured along a length of RPE and results in Figure 5 and Table 1 show the percentage of RPE containing deposits within the length of RPE analysed. BlamDs are variable in both *Chm^Flox^, Tyr-Cre+* and control mice, both in terms of frequency and extent. Control mice showed an average of 10±4% of RPE containing deposits, compared to 19±6% in *Chm^Flox^, Tyr-Cre+* mice (Fig. 5). However, only 3 out of 14 (21%) control mice aged between 5 and 13 months contained deposits along more than 10% of length of RPE compared to 7 out of 11 (64%) *Chm^Flox^, Tyr-Cre+* mice, demonstrating that loss of Rep1 in the RPE causes an increased likelihood of developing these deposits.

**Table 1:** Extracellular deposits in *Chm^flox^, TyrCre+* and littermate control mice.

	Age (months)	Gender	RPE length containingdeposits (%)*
**Flox**	6	M	0.1
	6	F	1.2
	6	F	9.3
	7	M	0.0
	7	M	2.1
	8	F	34.2
	10	F	42.4
	11	F	1.4
	12	M	0.9
	12	M	5.4
	13	M	2.6
	13	M	2.7
	13	M	27.6
**Flox, Tyr**	5	F	15.5
	5	M	0.0
	6	M	0.0
	7	M	0.0
	7	F	19.2
	7	M	25.8
	9	F	22.4
	12	M	67.3
	13	M	8.7
	13	M	19.3
	13	M	26.7

* percentage of the length of RPE analysed.

**Figure 6:**
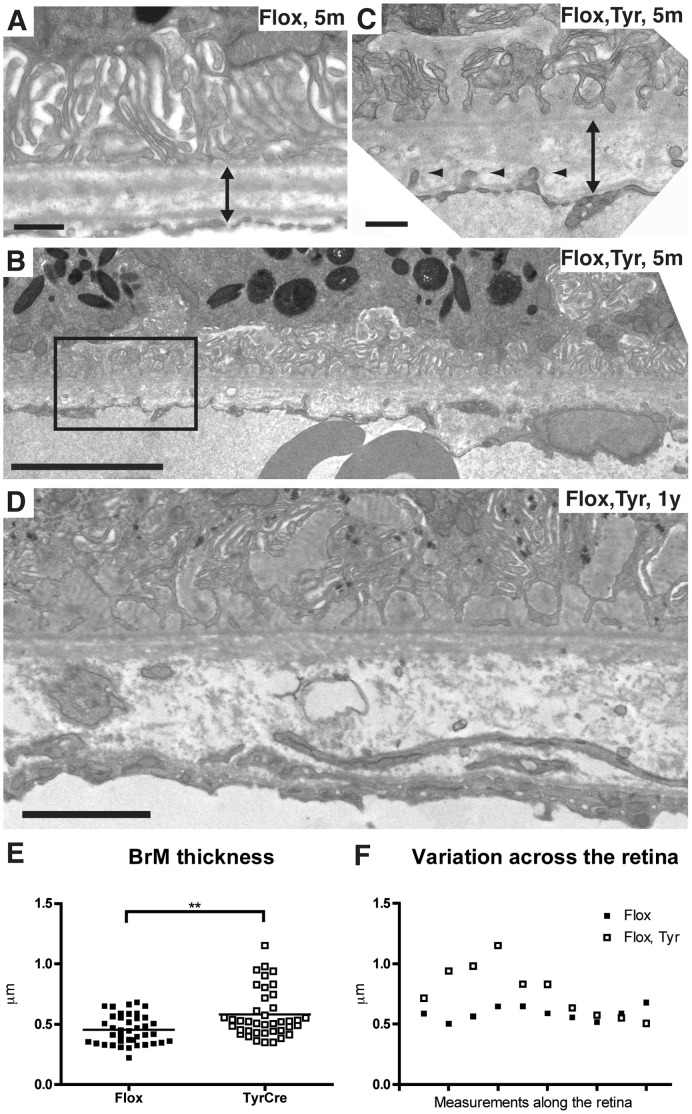
Thickening and abnormalities of Bruch’s Membrane in *Chm^Flox^*, *Tyr-Cre+* mice. Electron micrographs of 5-month old *Chm^Flox^* (A), littermate *Chm^Flox^*, *Tyr-Cre+* (B–C) and 1-year old *Chm^Flox^*, *Tyr-Cre+* mice (D). An enlargement of the box in B is shown in C. In *Chm^Flox^*, *Tyr-Cre+* mice BrM becomes thicker with time. Double arrows show BrM thickness, small arrowheads indicate endothelial cell protrusions into BrM. Scale bars: 0.5 µm (A and C), 2 µm (B), 1 µm (D). (E) BrM thickness was measured in four 7-month old *Chm^Flox^*, *Tyr-Cre+* mice (black square) and their littermate controls (grey dots). In each mouse 10 areas of retina were analysed. The two means are significantly different. ***P = 0.009. (F) Example of the variation of the measurements of BrM thickness along the retina for one *Chm^Flox^*, *Tyr-Cre+* mouse (black square) and its littermate control (grey dots).

Deposits were also observed beneath the basal lamina within BrM [Basal linear Deposits (BlinD)] (Fig. 6B–D). In *Chm^Flox^* mice, BrM is highly organized, approximately 0.5 µm thick, composed of the RPE basement membrane, the inner collagen layer, the elastic layer, the outer collagen layer and the basement membrane of the choriocapillaris (Fig. 6A). This organisation was lost in *Chm^Flox^, Tyr-Cre+* animals as young as 5-months and became even more dramatic by 1-year. BrM was thickened in some areas (Fig. 4D, 6B and 6C), containing vesicles and various membranes within it (Fig. 6B and 6D). In addition, endothelial cells from the choriocapillaris sent numerous protrusions towards BrM (Fig. 6B and 6C). The thickness of BrM was measured in four *Chm^Flox^, Tyr-Cre+* animals and their littermate controls. In control animals BrM was on average 0.45 µm ±0.02 thick compared to 0.58 µm ±0.03 in *Chm^Flox^, Tyr-Cre+* mice (Fig. 6E). Figure 6F illustrates how some areas of the retina were more affected than others in a *Chm^Flox^, Tyr-Cre+* mice. In this particular example, BrM is 1.5 to 2 times thicker than the control in half of the length of RPE analysed.

**Figure 7:**
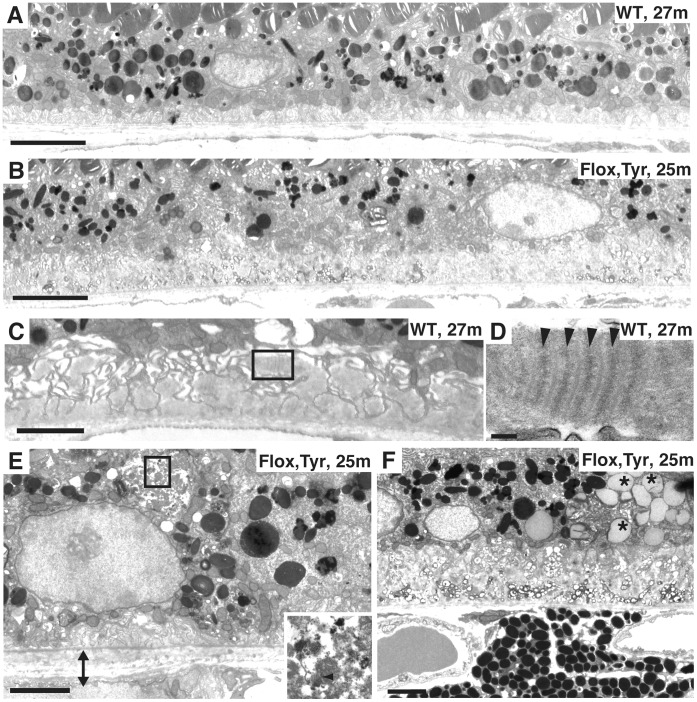
Basal deposits are present in old wild type mice but basal and intracellular deposits are much more extensive in old *Chm^Flox^, Tyr-Cre+* mice. Electron micrographs of a 27-month old WT mouse (A, C and D) and 25-month old *Chm^Flox^, Tyr-Cre+* mouse (B, E and F). Panel A shows the normal organisation of RPE cells found in some of the wild type eyecup. Panel B illustrates the extent of late BLamDs in old CHM animals, where the deposits are a third or up to half of the height of the RPE cells. Panel C shows that basal deposits can form in localised areas of the wild type eyecup. Panel D is an enlargement of the box in C, showing a banded pattern resembling long spaced collagen type VI in BLamDs. Panel E shows melanin, lipofuscin and membranes (arrowhead in inset) within the large cytoplasmic deposit (inset). The cell in panel F has accumulated a large number of lipid droplets (asterisks) and shows thick late BLamDs. Scale bars: 5 µm (A and B), 2 µm (C, E and F), 0.1 µm (D).

Irregularity in BIs and changes in BrM thickness were more severe in aged animals (older than 2-year) in both control and *Chm^Flox^, Tyr-Cre+* mice. In control mice RPE cells can exhibit a normal organisation (Fig. 7A) but disorganised BIs and early and late BLamDs can also be observed in areas of the eye (Fig. 7C). The striations in BLamDs (Fig. 7D) were more defined than in younger *Chm^Flox^, Tyr-Cre+* mice, and resembled banded collagen type VI. A combination of aging and loss of Rep1 resulted in an exacerbated phenotype such that in 2-year old *Chm^Flox^, Tyr-Cre+* mice, BrM was thicker in most places (Fig. 7B) and very large early and late BLamDs were frequently observed (Fig. 7B and 7F). Intracellular deposits were more frequent and larger in older *Chm^Flox^, Tyr-Cre+* mice and also contained lipid droplets (asterisks in Fig. 7F) and membranes resembling outer segment disks (arrowhead in inset of Fig. 7E).

## Discussion

In this study we examine the *in vivo* consequences of chronic defects in multiple membrane traffic pathways induced in the RPE. This has been achieved by deletion of the *Chm/Rep1* gene in pigmented cells, which results in poorly prenylated and consequently partly dysfunctional Rab GTPases, giving rise to multiple trafficking defects. Our demonstration that such trafficking defects can lead to the premature appearance of intracellular deposits of lipofuscin containing granules and melanolipofuscin, and extracellularly, thickening of BrM, and exuberant BLamDs, shows the importance of membrane traffic pathways in the maintenance of RPE health and tissue homeostasis.

### Loss of Rep1 in the RPE causes Partial Defects in Trafficking Pathways *in vivo*

We have focussed on two major specialised trafficking pathways in the RPE: the movement of melanosomes into the apical processes and the processing of phagocytosed rod outer segments. We have shown that the percentage of melanosomes in the apical processes of the RPE is reduced in the absence of Rep1 in the RPE. The movement of melanosomes into the apical processes is totally dependent on Rab27a function [6], [7] and so the small number of melanosomes that do access the apical processes in the absence of Rep1 demonstrates that some prenylated Rab27a must be present in these cells. Similarly, although phagosome degradation is clearly delayed in the absence of Rep1 in the RPE, the majority of the phagosomes are eventually degraded, suggesting only a partial dysfunction in this pathway.

Delayed phagosome degradation could result from delayed phagosome maturation and consequent delivery to the lysosome and/or lysosomal dysfunction. Phagosome maturation in other systems is a process dependent upon multiple Rab proteins that regulate sequential interactions with the endocytic pathway before the final Rab7-dependent fusion with the lysosome [17], [18], [19], [20], [21]. The huge and synchronised phagocytic load of the RPE may render it particularly susceptible to changes in function of individual phagosomal or endosomal Rab proteins. In addition, Gordiyenko et al. [10] found compromised lysosomal acidification following Rep1 depletion in cultured RPE cells, suggesting that the degradative capacity of the lysosome may be compromised.

### Loss of Rep1 Leads to the Premature Accumulation of Features Associated with aging in the RPE

*Chm^Flox^, Tyr-Cre+* mice exhibited lipofuscin containing granules and cytoplasmic deposits, uneven BI and accumulation of BLamDs and BlinDs within 6 months of age. The accumulation of lipofuscin intracellularly, uneven or enlarged basal infoldings and extracellular basal deposits are all features of aging in the eye [11], [12], [22], and we therefore propose that loss of Rep1 specifically in the RPE causes premature accumulation of changes associated with aging in these cells.

Which trafficking pathway defects might lead to these aging phenotypes? Although the majority of rhodopsin is eventually degraded in *Chm^Flox^, Tyr-Cre+* mice, delayed phagosome degradation is most likely responsible for the early accumulation of lipofuscin, the mixed vacuoles filled with lipofuscin and lipid droplets and membranes resembling disks from outer segments in *Chm^Flox^, Tyr-Cre+* mice. A delay in degradation of POS, rather than a complete block, is consistent with the phenotype of the *Chm^Flox^, Tyr-Cre+* mouse, as a complete block in degradation would lead to a more severe retinal phenotype, such as the one observed in the mcd/mcd mouse expressing an enzymatically inactive form of cathepsin D [23]. As mice have a much shorter life span than humans, the delay observed in the phagocytic pathway might not lead to degeneration of POS in the mouse but may contribute to the progressive degeneration of POS in CHM patients. Delayed phagosome processing and decreased lysosomal degradative capacity are unlikely to be due to reduced prenylation of Rab27a as lipofuscin and cytoplasmic deposits have not been reported in 7-month old *ashen* (Rab27a mutant) mice.

The accumulation of extracellular basal deposits in the CHM mouse indicates abnormal extracellular matrix (ECM) turnover resulting from either increased synthesis/secretion of ECM or reduced degradation. The RPE secretes multiple ECM components, including some components of BrM, and RPE cells from AMD donors have been found to secrete more ECM components than age-matched controls [24], suggesting that dysregulated ECM secretion could contribute to deposit formation. RPE cells express multiple matrix metalloproteinases (MMPs) and MMP inhibitors, and dysregulated traffic of these proteins could result in reduced extracellular matrix degradation. Reduced endocytosis from the basal border could lead to exaggerated basal infoldings and reduced lysosomal delivery of ECM. Defective lysosomal function (lysosomal storage diseases) and endocytic function (neurodegenerative diseases) are frequently associated with extracellular deposit formation (eg. amyloid plaques) and mice that express a mutant form of cathepsin D develop BlamDs [23]. Components of exosomes, lysosomes and autophagosomes have all been found in basal deposits, suggesting that secretion of undigested material may contribute to deposit formation [25]. It is also possible that secretion of factors from uveal melanocytes in the *Chm^Flox^, Tyr-Cre+* is impaired and this may affect thickening of BrM.

The *ashen* mouse has not been reported to prematurely develop extracellular deposits, suggesting that their formation in the CHM mouse is not due to poorly prenylated Rab27a and is likely to be the result of other or multiple Rab dysfunction. Together, these data suggest a relationship between defective degradative capacity inside the cell and accumulation of deposits outside the cell.

### Chronic Defects in Membrane Traffic Pathways could Play a Role in the Development of Features Associated with aging and AMD

AMD is a major cause of vision loss in the elderly population. In the ‘dry’ form of AMD RPE cells accumulate lipofuscin and their number and density in the macula is reduced. In addition, BrM thickens and BlamD, BlinD and drusen accumulate [26]. The more advanced ‘wet’ form of AMD is characterised by areas of RPE atrophy, neurodegeneration and choroidal neovascularisation. Despite the absence of a macula there are many mouse models of AMD that present some of the features of the human disease, including increased BlamD and BlinD, although in mice BlinD rarely progress to true drusen. Some mouse models also develop choroidal neovascularisation and retinal degeneration. The *Chm^Flox^, Tyr-Cre+* mice clearly present some features associated with ‘dry’ AMD, namely lipfuscin accumulation, BrM thickening and increased BlamD and BlinD. They do not develop choroidal neovascularisation, although they do develop endothelial cell protrusions into the basal lamina. Such protrusions have been observed in normal young eyes and do not necessarily lead to neovascularisation [27], but their increased presence in the *Chm^Flox^, Tyr-Cre+* mice suggests some changes in the choroidal vasculature.

Mouse transgenic models of AMD that form basal deposits include those caused by deletion/mutation of inflammatory genes (eg CFH, Ccr2, Ccl2), of genes related to oxidative stress (SOD2), metabolic pathway genes (ApoE, ApoB100) and intracellular proteases (cathepsin D, nephrilysin) [28]. Of these, the protease mutations have a clear direct link to deposit formation in that cathepsin D is important for the degradation of POS whilst nephrilysin is important for the degradation of beta amyloid, a component of drusen/basal deposits. Our mouse model thus contrasts with most existing models because the gene defect causes dysfunction of processes that could be primary causes of deposit formation ie defects in intra or extracellular degradation and secretion. Remarkably, very little attention has been paid to the role of membrane traffic in the formation of basal deposits. Although loss of Rep1 in the RPE is sufficient to increase the frequency of basal deposit formation and some changes in visual function [9] it is not sufficient to induce photoreceptor degeneration, as the neural retina is morphologically normal in the *Chm^Flox^, Tyr-Cre+* mice. In addition we haven’t observed obvious changes in the choroid of the *Chm^Flox^, Tyr-Cre*+ mouse, suggesting that the absence of REP1 in the RPE and uveal melanocytes does not have a major impact on the choroid, at least in the life span of a mouse. Similarly we cannot exclude the possibility that the loss of REP1 in uveal melanocytes might contribute to the RPE pathology in *Chm^Flox^, Tyr-Cre+* mice.

We previously showed that loss of Rep1 in the photoreceptors and the RPE caused a more rapid degeneration of photoreceptors than loss of Rep1 in photoreceptors alone [9], suggesting that chronic membrane traffic defects in the RPE can make the photoreceptors more susceptible to other insults. The similarity between the phenotype of the *Chm^Flox^, Tyr-Cre+* mice and changes associated with aging and early AMD pathogenesis suggest that these mice may allow the identification of trafficking pathways involved in these pathological changes.

## Supporting Information

Figure S1 **Pigmentation of uveal melanocytes in** ***Chm^Flox^, Tyr-Cre*** **+ mouse.** Electron micrographs of the choroids and RPE of 1-year old *Chm^Flox^* (A and C) and *Chm^Flox^, Tyr-Cre^+^* (B and D) mice. Panel C and D show high magnification images of the choroid of both mice. White asterisks indicate melanosomes of various sizes and black asterisks indicate where melanosomes have fallen out of the sections, (M) Mitochondria, (N) Nucleus. Scale bars: 10 µm (A, B), 1 µm (C, D).
(TIF)
