# Correction: Conditional Ablation of the Choroideremia Gene Causes Age-Related Changes in Mouse Retinal Pigment Epithelium

**DOI:** 10.1371/annotation/83a88285-e6a0-41fb-ae67-4315c21e5090

**Published:** 2013-05-06

**Authors:** Silène T. Wavre-Shapton, Tanya Tolmachova, Mafalda Lopes da Silva, Clare E. Futter, Miguel C. Seabra

There was an error in the name of the third author in the citation. The correct citation is:

Wavre-Shapton ST, Tolmachova T, Lopes da Silva M, Futter CE, Seabra MC (2013) Conditional Ablation of the Choroideremia Gene Causes Age-Related Changes in Mouse Retinal Pigment Epithelium. PLoS ONE 8(2): e57769. doi:10.1371/journal.pone.0057769 

