# Supplementary figures and images for: Conditional Ablation of the Choroideremia Gene Causes Age-Related Changes in Mouse Retinal Pigment Epithelium
Source: PLoS One. 2013 Feb 27;8(2):e57769. doi: 10.1371/journal.pone.0057769 (PMC3584022; doi:10.1371/journal.pone.0057769)

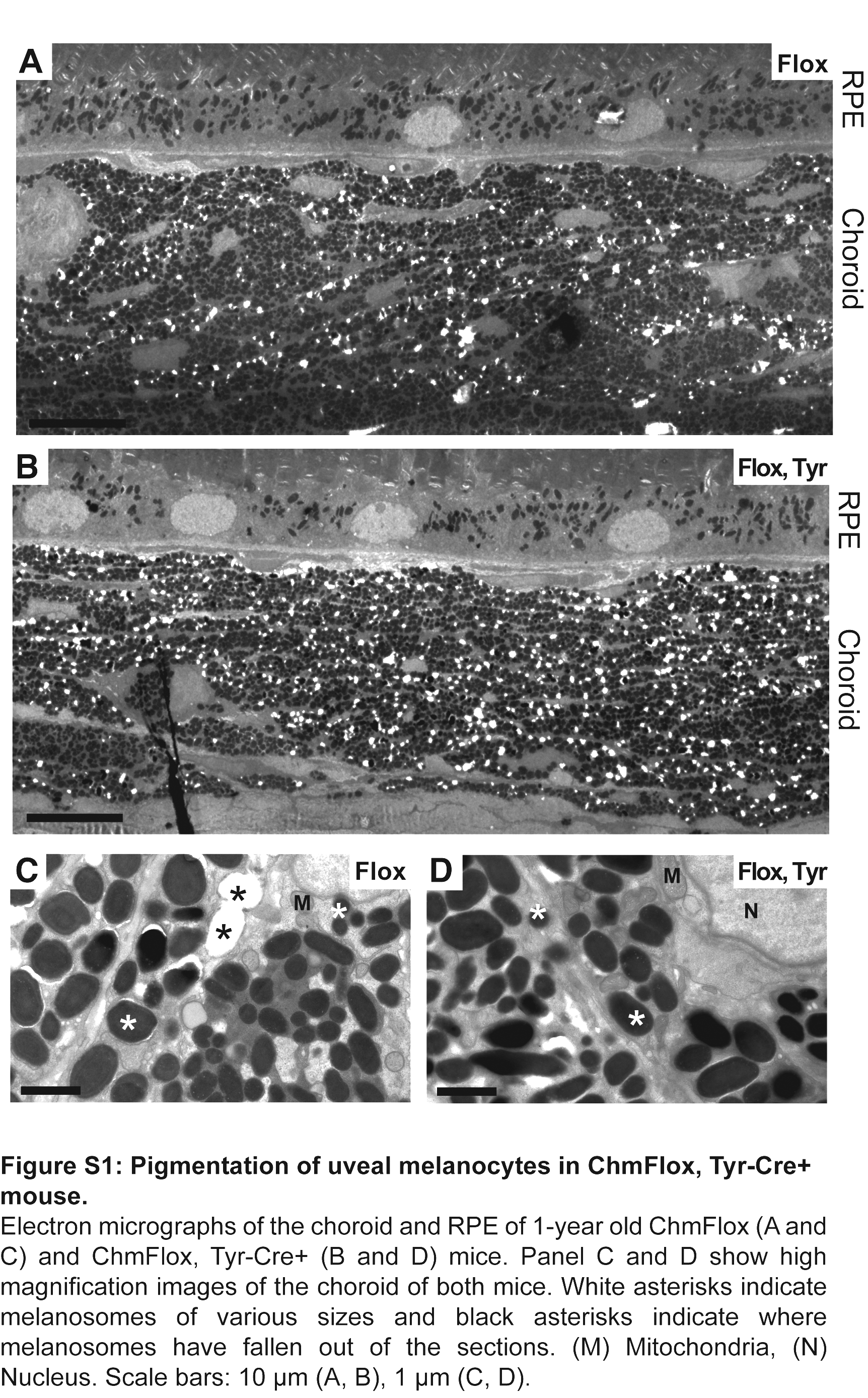

Supplement: Figure S1 — Pigmentation of uveal melanocytes in ChmFlox, Tyr-Cre + mouse. Electron micrographs of the choroids and RPE of 1-year old ChmFlox (A and C) and ChmFlox, Tyr-Cre+ (B and D) mice. Panel C and D show high magnification images of the choroid of both mice. White asterisks indicate melanosomes of various sizes and black asterisks indicate where melanosomes have fallen out of the sections, (M) Mitochondria, (N) Nucleus. Scale bars: 10 µm (A, B), 1 µm (C, D). (TIF) [file pone.0057769.s001.tif]
